# Extracorporeal Shockwave Therapy for Spasticity in Children With Cerebral Palsy: A Literature Review of Treatment Protocols and Outcome Measurement Parameters

**DOI:** 10.7759/cureus.84260

**Published:** 2025-05-16

**Authors:** Mohammed A Khan, Nada M Aljohani, Saad A AlSehemi, Salma A Abdelhamid, Omar A Alahmadi, Abdulmajeed Y Altohami, Abdullah A Shweikan, Mohammad K Aljohani

**Affiliations:** 1 Physical Therapy, Physioplans Physical therapy and Rehabilitation Centre, Medina, SAU; 2 Physical Therapy, King Salman Medical City, Medina, SAU; 3 Physical Therapy, Medical Rehabilitation Hospital, Medina, SAU

**Keywords:** cerebral palsy (cp), follow-up visit, functional outcomes, modified ashworth scale, muscle spasticity, parameters, radial extracorporeal shockwave therapy, range of motion (rom)

## Abstract

This literature review investigates the therapeutic potential of radial extracorporeal shockwave therapy (rESWT) for managing spasticity in children with cerebral palsy (CP), with a focus on its clinical efficacy, safety profile, and implications for functional rehabilitation. Synthesizing findings from six randomized controlled trials, the review reveals that rESWT consistently produces statistically significant reductions in spasticity, as measured by the Modified Ashworth Scale (MAS), along with notable improvements in gross motor function, joint range of motion, gait symmetry, plantar contact area, and manual dexterity. These benefits were maintained for varying durations, with some studies demonstrating sustained effects for up to 12 weeks post-treatment. Importantly, rESWT was well-tolerated, with no serious adverse events reported across all included studies, highlighting its safety and feasibility in pediatric populations.

Despite the consistent reporting of positive outcomes, considerable heterogeneity exists in treatment protocols, target muscle groups, outcome measurement timing, and reporting of participant characteristics, which complicates direct comparisons and limits the generalizability of findings. The review also emphasizes the need for incorporating multidimensional outcome domains, including those related to posture, balance, and gross motor performance, in future studies to better reflect real-world functional gains. Although promising, the current evidence base remains constrained by methodological inconsistencies and limited use of objective assessment tools. To optimize clinical application, future research should aim to standardize rESWT protocols, extend follow-up periods, and incorporate validated biomechanical and functional performance assessments. Collectively, these findings support rESWT as a non-invasive, clinically effective, and safe adjunct to conventional therapies in pediatric neurorehabilitation, with the potential to enhance both motor and functional outcomes in children with CP.

## Introduction and background

Cerebral palsy (CP) is recognized as a non-progressive neurological disorder stemming from damage to the immature brain, frequently resulting in a spectrum of motor disabilities [1,2]. Among the various motor impairments associated with CP, spasticity stands out as a primary concern for a significant majority of affected individuals [2,3]. This condition, affecting approximately 80% of children diagnosed with CP, is characterized by a velocity-dependent increase in muscle tone, exaggerated tendon reflexes, and an overall hyperexcitability of the stretch reflex [4,5]. Spasticity in children with CP can result in a series of difficulties, including persistent pain, impaired motor function, limited range of motion, and reduced ability to participate in daily activities, ultimately affecting their overall quality of life [6,7]. The widespread occurrence and debilitating effects of spasticity pose a considerable problem in clinical practice, underscoring the crucial necessity for ongoing research into effective treatment options in rehabilitation.

Extracorporeal Shockwave Therapy (ESWT) has emerged as a promising non-invasive intervention for spasticity in CP, requiring rigorous research to refine and enhance management protocols [8-10]. ESWT involves the delivery of high-amplitude, short-duration mechanical energy pulses, or shock waves, which are externally generated and directed toward target tissues to exert therapeutic effects [11]. A non-invasive, non-pharmacological, and generally low-risk intervention, ESWT is being increasingly investigated for its potential to alleviate spasticity in individuals with CP [12,13]. ESWT is delivered using two main techniques: radial shockwave therapy (rESWT) and focal shockwave therapy (f-ESWT), which differ in their generating mechanisms, physical properties, and tissue penetration levels [14-15]. Specifically, radial ESWT (rESWT) is commonly preferred in the management of muscle spasticity, owing to its predominantly superficial effect on soft tissues [16-18].

ESWT involves the transmission of high-energy acoustic pulses that rapidly increase in pressure and are followed by a brief phase of negative pressure or tensile force [19,20]. This biphasic waveform can exert therapeutic influence on both surface-level and deeper tissues, depending on the delivery method applied [21]. Focused ESWT generates sharply concentrated waves capable of reaching tissue depths up to 12 cm, making it suitable for treating large or deep muscle structures [22]. Conversely, radial ESWT produces waves that rise more gradually and disperse over a broader area, typically affecting tissues within a 3 to 4 cm depth [23].

While the definitive pathways through which ESWT exerts its effects on reducing muscle spasticity remain to be clarified, a number of physiological models have been proposed to explain its potential therapeutic action [24]. The suggested mechanisms include alterations in muscle mechanical properties, an increase in local nitric oxide production, vibratory effects on tendons that modulate neuromuscular control, a reduction in alpha motor neuron excitability, and transient modifications in peripheral nerve conduction [25-29]. Spasticity in CP is caused by non-progressive damage to the developing brain, typically involving the motor cortex, which results in lesions to the upper motor neurons [30]. This damage creates an imbalance in the excitatory and inhibitory signals of the central nervous system, leading to increased reflex activity and excessive muscle contraction [31]. A primary molecular factor in this process is the downregulation of potassium-chloride cotransporter 2 (KCC2), which disrupts chloride homeostasis and interferes with inhibitory neurotransmission in motor neurons. [32-34]. In clinical practice, spasticity is characterized by heightened resistance to passive movement, involuntary muscle spasms, contractures, and skeletal deformities, which collectively hinder mobility, restrict daily functioning, and negatively affect quality of life. [35,36].

Common approaches to managing spasticity include physical therapy, stretching, orthoses, pharmacological treatments, Botulinum toxin type A injections, and orthopedic or neurosurgical procedures [37-41]. However, these methods are often constrained by adverse effects, invasiveness, or high costs. In contrast, ESWT offers a more adaptable and minimally invasive alternative. Standardizing ESWT treatment protocols is critical for validating its role as an evidence-based intervention for spasticity in CP.

This literature review provides a comprehensive synthesis of randomized controlled trials on ESWT for spasticity management in children with CP. It focuses on identifying optimal protocol parameters such as energy flux density (EFD), frequency, duration, and total number of shocks, and evaluating the most appropriate timing for outcome measurements to achieve sustained reductions in spasticity. The central research question is: What is the optimal protocol of ESWT (in terms of frequency and total number of shocks) and the most appropriate timing for outcome measurements to achieve sustained reduction in spasticity in children with CP? By comparing outcomes across studies, the review aims to identify treatment trends, examine the impact of outcome timing on effectiveness, and assess functional improvements such as gait.

Due to the widespread impact of spasticity on children with CP, optimizing ESWT protocols is crucial. As a non-invasive, drug-free option, ESWT offers distinct benefits over traditional treatments. Fine-tuning treatment parameters and timing is essential for maximizing its efficacy and ensuring consistent outcomes across clinical settings.

Materials and methods

Search Strategy

A comprehensive literature search was conducted to identify studies examining the effects of ESWT on spasticity in children with CP. Three electronic databases were searched: PubMed Central, the Cochrane Library, and the Physiotherapy Evidence Database (PEDro). The search was limited to articles published in English from inception through April 2025. Table 1 provides a summary of the database search results. Filters were applied to restrict results to human studies and clinical trials. This strategy aimed to capture a broad range of randomized controlled trials evaluating the effects of ESWT on spasticity in pediatric populations.

**Table 1:** Summary of Database Search Results: Number of Studies and Search Strings Used Across PubMed, Cochrane, and PEDRo

Database	Number of Studies	Search Strings
PubMed	7	("Cerebral Palsy"[MeSH Terms] OR "Cerebral Palsy") AND ("Extracorporeal Shock Wave Therapy"[MeSH Terms] OR "Extracorporeal Shock Wave Therapy" OR "Shockwave Therapy" OR "ESWT") AND ("Spasticity"[MeSH Terms] OR "Spasticity" OR "Muscle Spasticity") AND ("Randomized Controlled Trial"[Publication Type] OR "Randomized Controlled Trial" OR "RCT") AND ("Child"[MeSH Terms] OR "Pediatric" OR "Adolescent" OR "Children")
Cochrane	9	(cerebral palsy) AND ("extracorporeal shock wave therapy" OR "shockwave therapy" OR ESWT) AND (spasticity OR "muscle spasticity") AND ("randomized controlled trial" OR RCT) AND (child OR children OR pediatric )
PEDro	7	Cerebral Palsy Extracorporeal Shock Wave Therapy
Total Studies	23	

Study Selection

**Figure 1:**
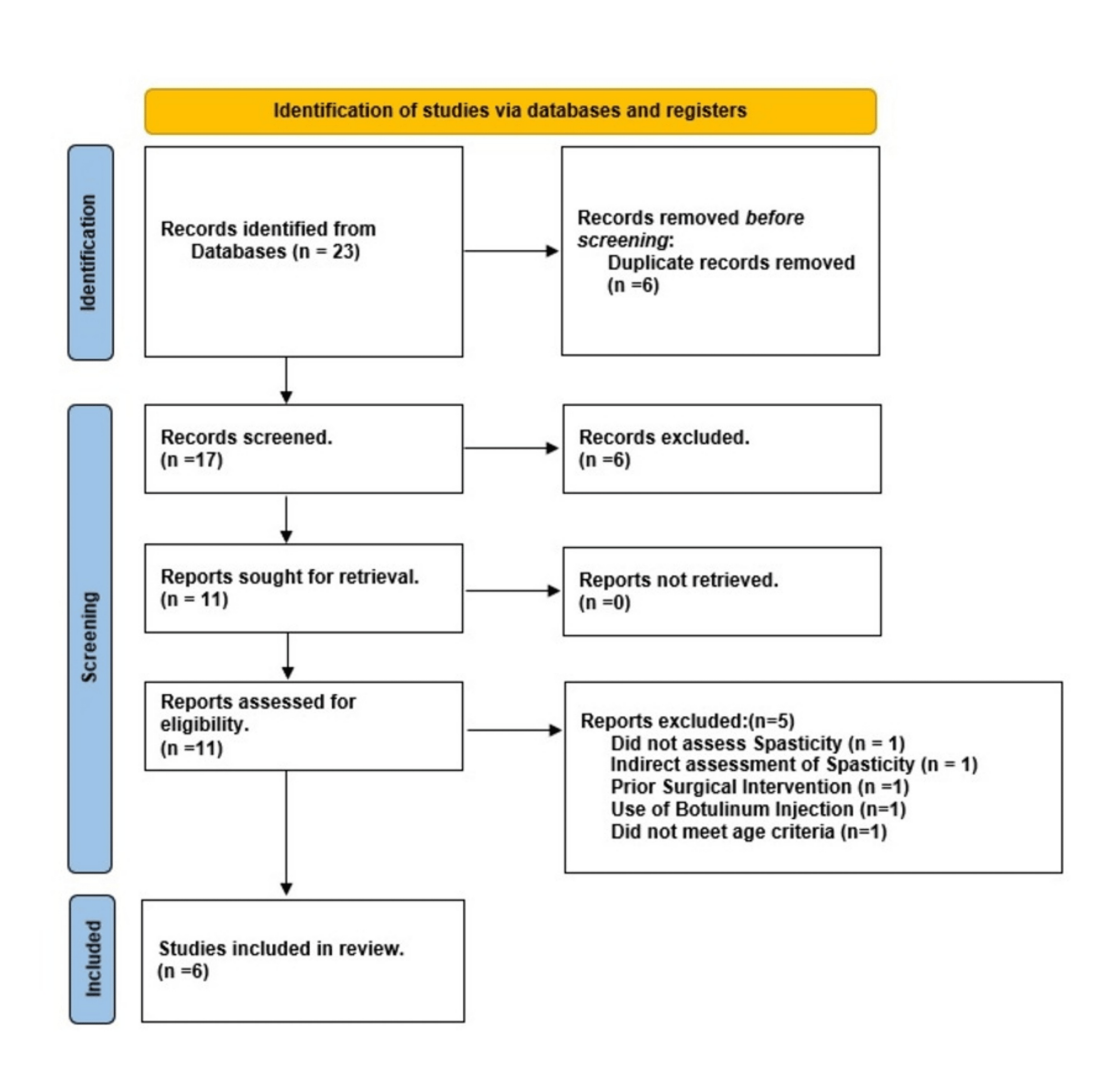
PRISMA Flow Diagram Flowchart illustrating the study selection process for the literature review, including identification, screening, eligibility assessment, and inclusion of studies on extracorporeal shockwave therapy (ESWT) for spasticity in children with cerebral palsy.

A total of 23 studies were initially identified through the search, conducted in accordance with the Preferred Reporting Items for Systematic Reviews and Meta-Analyses (PRISMA) guidelines [42]. After removing 6 duplicates, 17 studies remained for screening. Of these, six studies were excluded based on title and abstract review. The remaining 11 full-text articles were assessed for eligibility. Based on the predefined inclusion and exclusion criteria, six studies were included in the final analysis, while five were excluded for not meeting the eligibility requirements. Figure 1 illustrates the study selection process following the PRISMA flow diagram. In total, six studies comprising randomized controlled trials (RCTs) met the inclusion criteria and were included in this review. These studies evaluated the effects of ESWT on spasticity in children with CP.

Inclusion Criteria

For this review, eligible studies were those that examined the effects of ESWT on spasticity in children with CP. The studies included participants aged between 5 and 15 years who had a diagnosis of spastic diplegia or hemiplegia, with spasticity graded between 1 and 3 on the Modified Ashworth Scale (MAS) or assessed using the Australian Spasticity Assessment Scale (ASAS). The severity of motor impairment was assessed using the Gross Motor Function Classification System (GMFCS), and children were ideally classified within levels I to III. Studies were included even if GMFCS levels were not reported, as long as other inclusion criteria were met. The intervention had to involve ESWT, either radial (rESWT) or focused (fESWT), with clearly defined parameters, such as frequency and the number of shocks delivered. Studies that evaluated different pulse counts without a comparator group were also considered. Comparator groups, when applicable, could include no intervention, therapeutic exercises, placebo, or sham ESWT. Eligible studies were required to report a reduction in spasticity, assessed through the MAS or ASAS, and preferably included additional outcome measures like range of motion or gait improvements.

Exclusion Criteria

Studies were excluded from the review if they involved combined treatments, such as Botox injections or surgical interventions, as these were beyond the scope of this investigation, which focused exclusively on the impact of ESWT on spasticity. Additionally, non-randomized studies or those lacking a comparator group were excluded unless the primary focus was on evaluating different pulse counts in ESWT. These criteria ensured that only studies specifically investigating the effects of ESWT on spasticity in children with CP were included.

Risk of Bias Assessment

The methodological quality of the included studies was assessed using the Physiotherapy Evidence Database scale (PEDro,1999) [43]. The PEDro scale is a widely recognized tool for evaluating the quality of clinical trials, particularly in the field of physiotherapy and rehabilitation [44-46]. It consists of 11 items that assess key methodological aspects such as randomization, allocation concealment, blinding, and statistical analysis. Each study was independently scored based on the PEDro scale, with the first item (eligibility criteria specified) excluded from the final score. The remaining 10 items contribute to a total score out of 10, with a score of 6 or above considered indicative of high methodological quality. A summary of these studies, including interventions, study design, and outcome measures, is presented in Table 2. The PEDro scores for each included study are presented in Table 3.

**Table 2:** Summary and Baseline Characteristics of the Included Studies RCT: Randomized controlled trial; ASAS: Australian Spasticity Assessment Score; rESWT: Radial extracorporeal shockwave therapy; GMFM: Gross motor functional measurement; TSMC: Trost selective motor control; SLST: Single leg sit to stand; MAS: Modified Ashworth Scale; rSWT: Radial shock wave therapy; MHC: Modified house function classification; SD: Standard deviation

Sl. No	Author(s) & Year	Study Design	Participants	Age	CP Type	GMFCS Level	Spasticity Level and Assessment Tool	Intervention	Parameters	Comparator	Muscle Group	No. of Sessions	Outcomes Measured	Follow-up Period	Pedro Score
1	Wardhani et al., 2022 [47]	RCT	13	5-14	Diplegic	Not Mentioned	ASAS	rESWT	Group 1: Shocks:500, Pressure 1.5 bar, Frequency 4 Hz, Energy flux density: 0.1 mJ/mm^2^, Group 2 Shocks: 1000 Pressure 1.5 bar, Frequency: 4 Hz, Energy flux density: 0.1 mJ/mm^2^, Group 3 Shocks:1500, Pressure:1.5 bar, Frequency: 4 Hz, Energy flux density: 0.1 mJ/mm^2^, Group 4 Shocks: 2000, Pressure: 1.5 bar, Frequency: 4 Hz, Energy flux density: 0.1 mJ/mm^2^	None	Hamstring	1 session per week	ASAS	2 weeks and 4 weeks	5/10
2	Wardhani et al., 2022 [48]	RCT	14	5-12 years			ASAS	rESWT	Shocks: 1500, Pressure: 1.5 bar, Frequency: 4 Hz, Energy flux density: 0.1 mJ/mm^2^	rESWT + Sham rESWT	Gastrocnemius muscles	1 session per week	ASAS	4,8, and 12 weeks	5/10
3	Emara et al., 2022 [49]	RCT	31	7-9years	Not mentioned	Level I-II	1 - 1+ MAS	rESWT + Traditional Exercises	Shocks: 1500, Pressure: 1.5 bar, Frequency: 4 Hz; Energy flux density: 0.030 mJ/mm^2^	Traditional exercises	Calf muscle	1 session per week	Biodex dynamometer, GMFM-88-dimension D and E, TSMC, SLST, MAS,	12 weeks	7/10
4	Allam et al., 2021 [50]	RCT	34	5-7 years	Diplegic	Not mentioned	1 - 2 MAS	Traditional physiotherapy Program + ESWT	Shocks: 1200, Pressure: Not mentioned, Frequency: 4 Hz, Energy flux density: 0.12 mJ/mm^2^	Traditional physiotherapy Program	Knee flexors and hip adductors	12 sessions (1 session per week)	MAS, kinematic gait parameters	12 weeks	7/10
5	Farhan et al., 2019 [51]	RCT	32	5-15 years	Hemiplegic	Not mentioned	+1 – 3 MAS	Exercise program + ESWT	Shocks: 800- 2000, Pressure: Not mentioned, Frequency: 10 Hz, Energy flux density: 0.12 mJ/mm^2^, 0.03 mJ/mm^2^	Exercise program	Flexors of the Forearm and Interosseus muscles in the hand	8 sessions (1Session per week)	MAS, MHC	8 weeks	6/10
6	El-Shamy et al., 2014 [52]	RCT	30	Study group: mean age 6.93 years (SD 0.8), Control group: mean age 6.8 years (SD 0.7)	Hemiplegic	Not mentioned	Spasticity score mean value: Control group 2.27 (0.56), Study group 2.34 (0.48) MAS.	Conservative physiotherapy + ESWT	Shocks: 1500 Pressure 1.5 bar Frequency: 5Hz. Energy flux Density: 0.030 mJ/mm^2^	Conservative physiotherapy	Gastrocnemius muscles and the soleus muscle	Total 12 sessions (1 session per week)	MAS, 3D gait analysis	12 weeks	6/10

**Table 3:** Methodological Quality of the Studies

Author	Year	1	2	3	4	5	6	7	8	9	10	11	Total Score
Wardhani et al. [47]	2022	Yes	1	0	1	0	0	1	1	0	1	0	5
Wardhani et al. [48]	2022	Yes	1	0	1	1	0	1	0	0	1	0	5
Emara et al. [49]	2022	Yes	1	1	1	1	0	1	1	0	1	0	7
Allam et al. [50]	2021	Yes	1	0	1	1	0	1	1	0	1	1	7
Farhan et al. [51]	2019	Yes	1	0	1	0	0	1	1	0	1	1	6
El-Shamy et al. [52]	2014	Yes	1	1	1	0	0	1	1	0	1	0	6

## Review

This review included six randomized controlled trials (RCTs) that investigated the effects of rESWT on spasticity in children with CP [47-52]. All studies reported statistically significant improvements in spasticity following treatment, with some also demonstrating enhanced functional outcomes such as improved motor control, gait, and range of motion.

Spasticity reduction

Across all studies, rESWT led to a measurable reduction in muscle spasticity. Two studies used the ASAS as primary outcome measures [47,48], while four studies utilized the MAS [49-52]. Studies consistently demonstrated significant improvements following treatment. For instance, Allam et al. and El-Shamy et al. reported statistically significant reductions in MAS scores between the treatment and control groups post-treatment (P = 0.009 and P = 0.0003, respectively), in favor of the treatment groups [50,52]. Additionally, both studies observed significant within-group reductions in spasticity following the intervention [50,52]. Emara et al. and Farhan et al. observed similar benefits in spasticity, with both studies reporting statistically significant improvements (P < 0.01) [49,51]. Although Wardhani et al. found no significant intergroup differences among the four shock-dose groups, all groups demonstrated improvement in ASAS scores [47].

Session frequency and dose response

Two studies investigated the impact of session frequency and shock dose [47,48]. Wardhani et al. evaluated different shock doses (500 to 2000 pulses) and found that while all doses were effective, higher doses did not produce proportionately greater effects, suggesting a non-linear dose-response relationship [47]. In a subsequent study, the same authors compared three versus five sessions and observed no statistically significant differences in outcomes between the two groups at 4-, 8-, and 12-weeks post-treatment, suggesting a possible threshold effect [48].

Energy flux density and protocol variability

Most studies included in this review reported the EFD, which is a critical parameter in ESWT, with values ranging from 0.1 to 0.12 mJ/mm², corresponding to low-to-moderate energy levels [47-52]. Despite the variation in reported values across studies, all demonstrated significant reductions in spasticity, suggesting therapeutic benefits even at lower EFD levels. The variation in reporting underscores the need for more consistent documentation of treatment parameters in future research to allow better comparability.

Functional outcomes

Four studies reported additional functional outcomes beyond spasticity reduction [49-52]. Emara et al. and Allam et al. observed improvements in gross motor function (GMFM-D/E) and plantar surface area [49,50]. Furthermore, Allam et al. and El-Shamy et al. demonstrated enhanced gait characteristics, including increased stride length and improved symmetry, as measured by three-dimensional gait analysis using kinematic and spatiotemporal parameters [50,52]. Farhan et al., who focused on upper limb spasticity in children with hemiplegic CP, reported functional improvements in hand control (as measured by the Modified House Classification), underscoring the broader therapeutic potential of ESWT beyond lower limb applications [51]. Their findings highlight the role of ESWT in improving upper limb function, thereby supporting greater independence in daily tasks, including the effective use of assistive devices like walkers or canes.

Follow-up and duration of effects

Follow-up durations varied across studies, ranging from 2 weeks to 12 weeks [47-52]. Wardhani et al. reported follow-ups at 2 weeks and 4 weeks for the first study and at 4, 8, and 12 weeks for the second study [47,48]. Emara et al. and Allam et al. reported follow-ups at 12 weeks [49,50], Farhan et al. followed up at 8 weeks, and El-Shamy et al. reported follow-up at 12 weeks [51,52]. These studies generally found that improvements in spasticity were sustained throughout their respective follow-up periods.

Safety and tolerability

None of the included studies reported any major adverse events associated with rESWT. The available evidence reinforces the excellent safety and tolerability profile of rESWT in pediatric populations, supporting its use as a non-invasive therapeutic option for managing spasticity in children [47-52]. As summarized in Table 4, the comparative insights across the included studies reveal key findings regarding protocol parameters, spasticity reduction, shock dose effectiveness, and safety.

**Table 4:** Summary of Key Comparative Insights Across Included Studies

Reference	Aspect	Findings
Wardhani et al., 2022 [47], Wardhani et al., 2022 [48], Emara et al., 2022 [49], Allam et al., 2021 [50], Farhan et al., 2019 [51], El- Shamy et al., 2014 [52]	Protocol parameters	Shock doses ranged from 500 to 2000; session frequency varied (1–12 sessions), EFD ranged from 0.01 to 0.12 mJ/mm² when reported.
Wardhani et al., 2022 [47], Wardhani et al., 2022 [48], Emara et al., 2022 [49], Allam et al., 2021 [50], Farhan et al., 2019 [51], El- Shamy et al., 2014 [52]	Spasticity reduction	Consistently significant across all studies (MAS/ASAS)
Wardhani et al., 2022 [47]	Shock dose	No linear dose response; even lower doses are effective
Wardhani et al., 2022 [48]	Session frequency	3 vs. 5 sessions: similar outcomes, suggesting threshold effect
Emara et al., 2022 [49], Allam et al., 2021 [50], Farhan et al., 2019 [51], El-Shamy et al., 2014 [52]	Functional outcomes	Improved GMFM, gait, SLST, and MHC in treatment groups
Farhan et al., 2019 [51]	Muscle groups	Primarily lower limb: one study showed effectiveness in the upper limb
Wardhani et al., 2022 [48], Emara et al., 2022 [49], Allam et al., 2021 [50], El-Shamy et al., 2014 [52]	Follow-up duration	Benefits sustained for up to 12 weeks
Wardhani et al., 2022 [47], Wardhani et al., 2022 [48], Emara et al., 2022 [49], Allam et al., 2021 [50], Farhan et al., 2019 [51], El-Shamy et al., 2014 [52]	Safety	No adverse events were reported across all studies throughout the trial period.

Discussion

This literature review synthesized evidence from six RCTs examining the effects of rESWT on spasticity in children with CP [47-52]. All included studies reported statistically significant reductions in spasticity following the intervention, with improvements sustained for varying durations [47-52]. Among these, two trials specifically investigated the effects of session frequency and shock dosage [47,48]. Both found no additional benefit from higher frequencies or shock counts, suggesting a threshold effect wherein therapeutic gains plateau beyond a certain dosage [47,48]. This outcome highlights the possibility that minimal, well-structured therapeutic protocols can effectively contribute to meaningful clinical gains.

Wardhani et al. [48] implemented a structured follow-up protocol at 4, 8, and 12 weeks, demonstrating sustained improvements in both spasticity and functional outcomes over time. Similarly, Emara et al., Allam et al., Farhan et al., and El-Shamy et al. reported improvements not only in muscle tone but also in functional domains such as joint range of motion, plantar contact area, gait symmetry, hand function, and gross motor function [49-52]. These observations support the notion that rESWT may facilitate broader therapeutic aims, with effects that extend beyond neuromuscular modulation.

Across studies, the reported EFD, an essential therapeutic parameter, ranged from 0.1 to 0.12 mJ/mm², with frequencies typically set between 4 and 10 Hz [47-52]. These values align with low-to-moderate intensity protocols and appear effective for pediatric populations. However, inconsistencies in the documentation of EFD, shock count, and application techniques limit interpretability and emphasize the importance of standardization in future research.

Although the included trials generally report favorable outcomes, the heterogeneity in study methodologies limits the broader applicability of the results. Key sources of variation include the choice of target muscles, diversity in outcome assessments, and differences in participant profiles-especially in GMFCS levels. Additionally, few studies utilized objective biomechanical assessments (e.g., kinematic gait analysis, torque measurements), limiting deeper understanding of rESWT’s physiological impact [49,50,52].

A recent meta-analysis by Chang et al. [53] corroborates these findings, indicating that although reductions in spasticity measured by the MAS may diminish after one month, functional gains such as increased passive ankle range of motion and improved plantar contact can persist for up to three months. These results underscore the importance of evaluating functional mobility parameters alongside spasticity measures in future ESWT research.

Further supporting the review’s conclusions, Su et al. conducted a 12-week study using low-intensity ESWT (1500 pulses at 0.1 mJ/mm², 4 Hz) in children with CP and Rett’s syndrome, reporting significant improvements in spasticity and ankle range of motion in the CP subgroup [54]. Notably, they employed acoustic radiation force impulse (ARFI) ultrasonography to measure changes in muscle stiffness, demonstrating a reduction in muscle rigidity. This suggests that ESWT may alter the intrinsic biomechanical properties of spastic muscle tissue, not merely reduce neural hyperexcitability [54].

Additional insights are offered by de Roo et al., who conducted a systematic review of 12 RCTs evaluating ESWT in children with spastic CP [55]. Their analysis highlighted significant MAS score reductions and large effect sizes for passive range of motion, baropodometry, selective motor control, and gross motor function. Importantly, most studies described the intervention as painless or free from discomfort, reinforcing its safety profile [55].

Mirea et al. further confirmed ESWT’s rehabilitative value, demonstrating that a 3-session protocol (0.15 mJ/mm², 500 shocks per session, 10 Hz) led to a nearly one-point reduction in MAS scores, a 10-point improvement in GMFM-66, and a 14-point reduction in pain scores in children with diplegia or quadriplegia [56]. These results suggest that ESWT not only alleviates spasticity but also reduces pain and enhances functional capacity, reinforcing its role in comprehensive pediatric neurorehabilitation.

In summary, this review supports rESWT as a non-invasive, well-tolerated, and clinically effective intervention for managing spasticity in children with CP. The consistent absence of serious adverse events, along with its functional benefits across diverse motor domains, makes it a promising adjunct to conventional therapies. To enhance its clinical integration, future studies should focus on standardizing dosing parameters, particularly EFD, extending follow-up durations, and incorporating objective measurement tools. Moreover, functional and imaging-based assessments should be prioritized to capture the full scope of ESWT's therapeutic potential.

## Conclusions

This literature review highlights the growing body of evidence supporting rESWT as an effective, non-invasive treatment for reducing spasticity and enhancing functional outcomes in children with CP. The reviewed studies consistently demonstrated significant improvements in spasticity, joint range of motion, gait, and other functional measures following rESWT, with benefits sustained for weeks after treatment. Furthermore, the therapy’s safety profile was robust, with no serious adverse events reported, making it a promising adjunct in pediatric neurorehabilitation.

Despite these encouraging findings, variations in treatment protocols and outcome measurements underscore the need for more standardized and rigorous research to refine dosage parameters and assess long-term effects. The incorporation of objective measurement techniques, such as imaging-based assessments of muscle stiffness, would greatly enhance understanding of the underlying mechanisms of action and support the development of more personalized treatment plans.

Moving forward, well-designed studies with consistent protocols, comprehensive outcome measures, and extended follow-up periods are essential to establish rESWT as a cornerstone in the management of spasticity in CP, ultimately aiming to improve the quality of life for affected children.
